# Substance Use Outcomes For Sexual and Gender Minority Adults With a History of Adverse Childhood Experiences: A Scoping Review

**DOI:** 10.1016/j.dadr.2022.100129

**Published:** 2022-12-09

**Authors:** Brockton A. Dowling, Timothy J. Grigsby, Gregory J. Ziomek, Philip W. Schnarrs

**Affiliations:** a School of Medicine, West Virginia University, 64 Medical Center Drive, PO Box 9100, Morgantown, WV, 26506, USA; b Department of Social and Behavioral Health, University of Nevada, 4505 S. Maryland Parkway, Las Vegas, Nevada, 89154, USA; c Department of Psychiatry, Dell Medical School, The University of Texas at Austin, 1301 W 38th Street Suite 700, Austin, Texas, 78722, USA; d Division of Community Engagement and Health Equity, Department of Population Health, Dell Medical School, The University of Texas at Austin, 1601 Trinity Street, HDB 4.408, Austin, Texas, 78712, USA

**Keywords:** ACE, LGBT, alcohol, smoking, cannabis, substance use disorder

## Abstract

**Purpose:**

The impacts of Adverse Childhood Experiences (ACEs) on behavioral, mental, and physical health have been extensively investigated. As such, it is paramount to synthesize their quantified effects, especially within vulnerable populations. The goal of this scoping review was to collect, summarize, and synthesize existing research on ACEs and substance use (SU) in adult sexual and gender minority (SGM) populations.

**Methods:**

WebofScience, APA PsychInfo, LGBTQ+ Life (EBSCO), Google Scholar, and PubMed electronic databases were searched. We included reports published between 2014 and 2022 that assessed SU outcomes, ACEs in adult (18+) SGM populations, in the United States (US). We excluded those in which SU was not an outcome, measured community-based abuse or neglect, or investigated adulthood trauma. Data were extracted using the Matrix Method and categorized across three SU outcomes.

**Results:**

Twenty reports were included in the review. Nineteen employed a cross-sectional design and 80% focused on a single SGM group (transgender women, bisexual Latino men, etc.). Nine of 11 manuscripts found SU frequency and quantity were higher among ACE exposed participants. Three of four studies found ACE exposure to correlate with substance use problems and substance misuse. Four of five studies found ACE exposure to be correlated with substance use disorders.

**Conclusions:**

Longitudinal research is needed to comprehend the impact of ACE on SU within the diverse subgroups of SGM adults. Investigators should prioritize the use of standard operationalizations of ACE and SU to improve comparability across studies and include diverse samples from the SGM community.

## Highlights

• Adverse childhood experiences (ACE) impacts substance use behavior.
• Sexual and gender minorities are more likely to report ACEs.
• Sexual and gender minority subgroups showed adult substance use post ACE.
• Childhood sexual abuse was positively associated with substance use problems.

## 1 Introduction

Adverse childhood experiences (ACEs) are typically described as traumatic or stressful events that occur in household settings before the age of 18 that include childhood abuse and neglect, as well as challenges in the home such as exposure to domestic violence, caregiver substance use, or mental illness (Felitti et al., 1998). More than two decades ago, Felitti and colleagues (1998) documented the negative impacts of ACE exposure on adult health with greater exposure increasing risks for undesirable health outcomes. Exposure to any ACEs has been linked to higher odds of poor mental health (e.g., depression, anxiety, post-traumatic stress disorder), physical health (e.g., cancer, HIV), and behavioral health (e.g., substance use, aggression) outcomes. This relationship was graded with those reporting four or more ACE at the highest risk for any of those outcomes (Felitti et al., 1998). Moreover, exposure to more ACEs has been linked to increased risk of premature mortality (Jai & Lubetkin, 2020) and onset of chronic disease early in the life span (Sonu et al., 2019). In fact, one study found that exposure to 6 or more ACEs reduced average life expectancy by as much as 20 years (Brown et al., 2009).

The ACEs framework provides a useful model for understanding how exposure to trauma in childhood is associated with poor adult health (Hughes & Ostrout, 2020). Undergirded by a biopsychosocial approach (Wade & Halligan, 2017), the ACEs framework suggests that exposure to adversity during key points of human development alters neurodevelopment, (Sheridan & McLaughlin, 2020) leading to atypical developmental trajectories (Sachs-Ericsson et al., 2016; Szilagyi & Halfon, 2015) and causing psychological and biological dysfunction that persists over the life course (Agorastos et al., 2019). These dysfunctions can result in poor coping and increases in risk-taking and stress sensitivity (van Duin et al., 2019), further increasing risk for morbidity and mortality. Specific to biological changes, ACE exposure causes changes to the structure and function of neural-stress regulatory circuits such as the hippocampus and amygdala, heightening individuals’ attention toward threatening stimuli and increasing their stress sensitivity (Herzog & Schmahl, 2018). In addition, ACE exposure is also linked to worse mental health outcomes and psychiatric conditions through avoidant emotion focused coping (Sheffler et al., 2019). Taken together, there is strong evidence to show that exposure to ACEs have both biological and psychological consequences over the life course.

Sexual and gender minority (SGM) individuals - defined as lesbian, gay, bisexual, transgender, queer, questioning, and other SGM individuals who identify as something other than cisgender (corresponding biological sex and personal identity) and/or heterosexual - report greater ACE exposure compared to heterosexual adults (Austin et al., 2016). More specifically, past research has demonstrated that *sexual minority* (i.e, lesbian, gay, bisexual individuals versus heterosexual) individuals report greater ACE exposure overall and are more likely to report individual ACE items compared to heterosexuals (Andersen & Blosnich, 2013). Similarly, *gender minority* (i.e., transgender or non-gender conforming) individuals report greater ACE exposure overall and are more likely to report emotional abuse, as well as emotional and physical neglect compared to cisgender sexual minorities (Schnarrs et al., 2019). In addition, SGM individuals report frequent exposure to ACEs (Bond et al., 2021) and data show that emotional abuse and neglect are some of the most common ACEs reported by this population (Schnarrs et al., 2020, Schnarrs et al. 2022). Increased exposure to ACEs in this population is likely linked to discrimination and mistreatment related to heterosexism (Schnarrs et al., 2022). Heterosexism is a set of societal norms and beliefs that confer value on male-female sexual and romantic relationships (Herek, 1996), and includes societal and culturally engrained beliefs that only two genders (i.e., the gender binary) - men and women - exist, lead to devaluing non-heterosexual relationships and identities, as well as atypical gender identities and forms of gender expression (Saguy et al., 2021). Negative reactions to another's sexual- or gender- identity are particularly problematic when it occurs within the home. McGeough & Sterzing (2018) and Katz-Wise et al. (2014) observed caretakers withdraw resources, reject, and even commit violence against dependents and youth due to their being SGM. The varying degrees in response by a family not accepting a SGM family member, especially during youth, can contribute to an increasing number of ACEs above those experienced by individuals who are not SGM.

Nationally representative and community data have evidenced higher rates of substance misuse and substance use disorder (SUD) in SGM populations compared to cisgender and heterosexual peers (Medley et al., 2016; Substance Abuse and Mental Health Services Administration (SAMHSA), 2020); however, there is limited research identifying the role that ACEs play in explaining this disparity. Moreover, there are notable differences between minority subgroups, with bisexual men and women (Green & Feinstein, 2012) and gender minority individuals (Connolly & Gilchrist, 2020) reporting higher rates of substance use/misuse and SUD. Past work in this area has shown that victimization, especially during childhood (McCabe, 2020) is a potential explanatory factor associated with disparities in substance misuse and SUD.

Schneeberger et al. (2014) investigated the relationship between ACEs and multiple health outcomes in sexual minority adults. The paper was wide in scope, investigating rates and types of revictimization, dysfunctional behavioral adjustments, psychiatric symptoms, substance use, and other outcomes. The authors found that childhood sexual abuse (CSA) was the most commonly investigated measure of ACE and significantly correlated with substance use outcomes. The goal of the present review is to 1) update the field on the relationship between ACEs and multiple substance use outcomes since the Schneeberger et al. (2014) review, and 2) to include a review of the literature on the relationship between ACEs and substance use in gender minority populations.

## 2 Materials and Methods

This study followed the scoping review framework outlined by Arksey and O'Malley (2005) and the Preferred Reporting Items for Systematic Reviews and Meta-Analyses extension for Scoping Review (PRISMA-ScR) statement (Tricco et al., 2018) to map relevant research, summarize findings, and articulate knowledge gaps. This method is effective for analyzing emerging and specialized interests within research, well suited to explore ACEs and substance use outcomes in the SGM community. No protocol was registered for this study in advance for searching for relevant studies.

The primary search was conducted within WebofScience, APA PsychInfo, and LGBTQ+ Life (EBSCO) databases. The following search terms were used: outcomes (*alcohol, tobacco, cannabis, club drug, hard drug, illicit drug, prescription drug misuse, problem substance use, substance related negative consequences, drug abuse, drug dependence, substance-related disorders, substance use disorders*); predictors (*domestic abuse, physical abuse, sexual abuse, neglect, adverse childhood experiences, childhood maltreatment*); population (*LGBT, gay, lesbian, transgender, bisexual, sexual diverse, sexual minority, gender minority*). Boolean operators and truncation strategies were used to expand and then narrow the search for relevant articles with consultation with university academic staff members. Additionally, manual searches using general search terms and screening inquiry were undertaken to identify additional studies of relevance through Google Scholar and PubMed electronic databases.

### 2.1 Literature Search

**Fig. 1:**
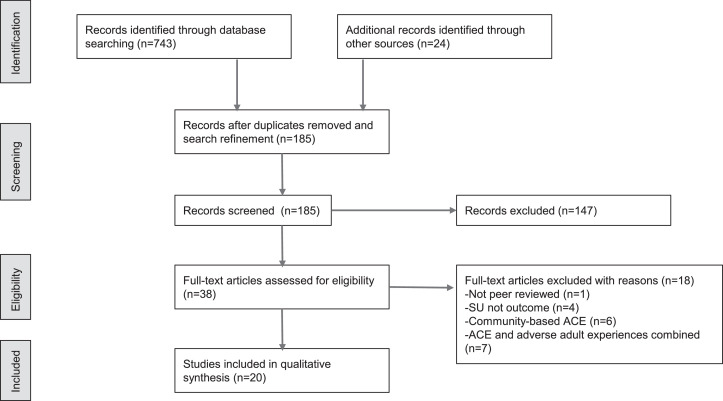
PRISMA flowchart of study selection

The primary search was conducted between November 2021 and February 2022 and captured relevant peer-reviewed empirical research studies published since the Schneeberger et al. (2014) review. Two reviewers independently reviewed the titles and abstracts of retrieved articles and screened for the following six inclusion criteria for peer-reviewed papers: 1) published between 2014 -2022, contained 2) a measure of substance use, misuse, or related substance use outcome, 3) a measure of adverse childhood experiences (as defined by Felitti et al., 1998) and the population of interest was 4) located in the United States, 5) aged 18 or older, and 6) included SGM persons. A third reviewer resolved disagreements and the research team reached unanimous consensus regarding disputes. Fig. 1 shows the process of study selection. The initial primary search produced 743 manuscripts. Specifically, PsychInfo produced 543 results, the search was further refined by selecting *relevance, human subjects*, and *peer-reviewed articles* on an advanced search tool. After this advanced search and removing duplicates within the primary search, 161 articles were retrieved. The manual search generated 24 results. After the title and abstract review, 38 potentially relevant articles were retrieved and were subject to a second round of full-text assessment by 2 reviewers. Of those, 18 studies were excluded from the final review (see Fig. 1). Twenty articles met the eligibility criteria and were included in the final review.

### 2.2 Data Extraction and Analysis

The Matrix Method (Garrard, 2017) was employed for extracting and tabling relevant information, providing an area to compare, contrast, and synthesize findings. The matrix produced with Microsoft Excel (Microsoft Corporation, Redman WA) included the last name of the lead author, publication year, study design, sample size, population, ACE measure name, ACE operationalization, ACE prevalence, outcome (substance type and behavior measured), substance measure name, type of analysis, and main findings pertinent to the research question. Within the matrix, articles were divided into three categorie*s* based on the operationalization of the outcome*: Substance Use Frequency and Quantity, Substance Use Problems and Misuse,* and *Substance Use Disorder* (Table 1). Substance use problems (also known as negative substance use consequences) are the proximal and deleterious effects of substance use and include physical, psychological, social, and behavioral harms such as blacking out, being unable to cut down use, and neglecting social responsibilities. Chronic patterns of substance use problems represent a behavioral pattern of substance misuse (Grigsby, 2021a). These three categories were selected to demonstrate the range of interest in the interactions between ACEs and substance use outcomes; specifically, from reported use to clinically significant disorders. The relevant information was first recorded into the table by a researcher, once completed a second researcher validated the information, and a third researcher was included to resolve disagreements.

**Table 1:** Summary of studies on ACE, Substance Use, Substance Misuse, and SUD in SGM populations

Author and Year	Sample demographics	ACE measure	Outcome measure	Main findings
*Substance Use Frequency and Quantity*				
Austin et al. (2016)	Study design: Cross-sectional Sample Size: 30,401 Population: LGB	Measure name: BRFSS-ACE module Operationalization: Cumulative ACE, Individual ACE ACE prevalence: 73.2% LGB report 1+ ACE; 42.4% report 3+ ACE	Outcome: Cigarette Use, Binge Drinking, Heavy Drinking Measure: Investigator Created Analysis: Multivariable Logistic Regression	While GB Adults more likely to report any ACE category compared to heterosexuals, there was no difference in cigarette use, binge drinking, or heavy alcohol use by sexual orientation after adjusting cumulative ACEs exposure.
Bertolino et al. (2020)	Study design: Cross-sectional Sample Size: 2,590 Population: MSM	Measure name: Adapted-ACE-Q (Felliti, 1998) Operationalization: Individual and any ACE exposure ACE prevalence: 1+ ACE = 79.7%	Outcome: Illicit substance use Measure: Investigator created Analysis: Log-binomial Regression	MSM exposed to any ACE were more likely to report illicit substance use. Illicit substance use prevalence was associated with individual ACE - psychological and physical abuse, exposure to mental illness or substance abuse and parental divorce. Sexual abuse, violence toward mother, and familial incarceration were not related to illicit substance use.
Giano et al. (2019)	Study design: Cross-sectional Sample Size: 156 Population: MSM	Measure name: ACE-Q (Felliti, 1998) Operationalization: Cumulative ACE ACE prevalence: M = 2.79	Outcome: Marijuana Use Measure: Investigator created Analysis: Linear Regression, Split-sample regression	Prevalence of ACEs did not differ between urban and rural MSM. Reported ACEs were significantly associated with marijuana use in urban MSM, but not in rural MSM.
Grigsby et al. (2021b)	Study design: Cross-sectional Sample size: 11,694 Population: SGM and non-SGM college students	Measure name: Adapted-ACE-Q (Felliti, 1998) Operationalization: Cumulative ACE ACE prevalence: HMA<30%, HMD<20%, P/CI>10%, PIPV>20%, CPA>20%, CVA>50%, CSA<20%	Outcome: Cigarette use, ENDS use, dual use frequency Measure: ACHA-NCHA Analysis: Logistic Regression	Every additional ACE reported was associated with an increased odds of cigarette, e-cigarette, and dual use. ACE-exposed SGM had significantly higher prevalence of past 30-day cigarette use compared to non-SGM college students.
Lee et al. (2020)	Study design: Cross-sectional Sample Size: 453 Population: SGM Adults	Measure name: ACE-Q (Felliti, 1998) Operationalization: Individual ACE ACE prevalence: Not Reported	Outcome: Tobacco use Measure: Investigator created Analysis: Logistic Regression	In unadjusted models, substance abuse in the home was associated with an increased odds of current cigarette smoking among bisexual and transgender or nonbinary participants. CPA was also associated with increased odds of cigarette smoking for bisexual participants and young adult (age 18-24) SGM participants.
Kecojevic et al. (2015)	Study design: Cross-sectional Sample Size: 191 Population: YMSM	Measure name: CTQ Operationalization: Individual ACE ACE prevalence: 65.5% CEA, 54.5% CPA, 37.8% CSA	Outcome: Prescription drug misuse, Illicit drug use Measure: SAMHSA, Investigator created Analysis: Poisson regression	CPA was significantly associated with higher prescription opioid misuse, but not prescription tranquilizer, prescription stimulant, or illicit drug use. CEA and CSA were not significantly associated with prescription drug misuse or illicit drug use.
Mattera et al. (2018)	Study design: Cross-sectional Sample Size: 148 Population: Bisexual Latino men	Measure name: Investigator created- CSA type Operationalization: Individual ACE ACE prevalence: 1+ CSA = 22%	Outcome: Polydrug use Measure: Investigator created Analysis: Logistic Regression	CSA was not significantly associated with past four-month polydrug use.
Paul & Monahan (2019)	Study design: Longitudinal Sample Size: 648 Population: SM (youth 1994 - adult 2008)	Measure name: Investigator Created: CVA, CSA, CPA Operationalization: Discrete ACE ACE prevalence: 67.3% SM, 48.1% Heterosexual reported 1+ child maltreatment	Outcome: Drugs and Alcohol Use Measure: Investigator created Analysis: Linear Regression	There was a significant difference in methamphetamine use between maltreated and non-maltreated SM adults. However, no differences were observed for prescription drug misuse, steroid use, marijuana use, cocaine use, hallucinogen use, or days intoxicated.
Reback et al. (2017)	Study design: Cross-sectional Sample Size: 139 Population: Trans women of color living with HIV	Measure name: ETI-SR: CSA Operationalization: CSA Exposed ACE prevalence: 1+ CSA= 27.3%	Outcome: Illicit substance use Measure: The Los Angeles Transgender Health Survey Analysis: Adjusted Multivariable Logistic Regression and Negative Binomial Regression	Independently, both analyses found CSA to be significant and positively associated with both drug use and the number of drugs used.
Scroggs & Love (2022)	Study design: Cross-sectional Sample Size: 1,852 Population: SGM Adults	Measure name: ACE-Q (Felliti, 1998) Operationalization: Cumulative ACE ACE prevalence: M = 3.22	Outcome: Past 6-month frequency Measure: Investigator created Analysis: Pairwise correlations	ACEs were weakly but positively associated with past 6-month cannabis, tobacco, alcohol, opioid, heroin, hallucinogen, and amphetamine use.
Suarez et al. (2021)	Study design: Cross-sectional Sample Size: 131 Population: Transmasculine adults	Measure name: ACE-Q (Felliti, 1998) Operationalization: Ordinal ACE, Individual ACE ACE prevalence: 4+ ACE = 45%	Outcome: Past 6-month binge drinking and substance use. Current cigarette use. Measure: Investigator created Analysis: Logistic Regression	The conditional probability of drug use ranged from 47-81% across categories of ACE. Reporting 4+ ACE was associated with an increased odds of substance use but ACE was not associated with binge drinking or cigarette use.
*Substance Use Problems and Misuse*				
Hughes et al. (2014)	Study design: Cross-sectional Sample Size: 1573 Population: Heterosexual and sexual minority women	Measure name: Wyatt Sexual Health Questionnaire- CSA; Investigator Created- CPA, Neglect Operationalization: Discrete ACE prevalence: CSA- 50%, CPA- 50%, Neglect-10% in SMW (includes mostly heterosexual)	Outcome: Hazardous Drinking Measure: Investigator Created Analysis: Logistic Regression	CSA in childhood was not associated with higher odds of hazardous drinking. But, victimization in childhood and adulthood was associated with an increased odds of hazardous drinking for sexual minority women.
Sizemore et al. (2021)	Study design: Cross-sectional Sample Size: 213 Population: Transgender women	Measure name: Investigator created Operationalization: CSA exposure ACE prevalence: 50.9%	Outcome: Alcohol use problems, Substance use problems Measure: SIP-AD, AUDIT Analysis: Moderated mediation regression model	Pairwise correlations showed a significant positive, weak correlation with substance use problems and a non-significant relationship with alcohol use problems. There was a significant three-way interaction between CSA, attachment anxiety, and attachment avoidance indicating those with high levels of attachment anxiety reported more substance use problems if they had a CSA history, those who were high in attachment avoidance, or high in both attachment anxiety and avoidance, had high levels of substance use problems regardless of CSA history. Findings suggest that secure attachment may also buffer against substance use problems for individuals with a CSA history.
Hall et al. (2021)	Study design: Cross-sectional Sample Size: 1,035 Population: MSM&TW	Measure name: Lesserman CSA History Operationalization: Discrete ACE prevalence: 26.3% report 1+ CSA event	Outcome: Cannabis Problems, Alcohol Problems, Stimulant Use Measure: CUDIT-R, AUDIT, Investigator Created items Analysis: Negative Binomial Regression	Pre-teenage forced touching and teenage touching were independently associated with alcohol use problems. An interaction between pre-teenage forced touching and teenage forced penetration was associated with increased alcohol problems. Teenage forced penetration was associated with cannabis problems. Moderation analysis showed participants who reported both pre-teenage penetration and teenage touching were less likely to report cannabis use problems compared to those who did not experience CSA. Teenage forced touching and teenaged forced penetration were associated with higher odds of using stimulants.
Yuan et al. (2014)	Study design: Cross-sectional Sample Size: 294 Population: Urban Two-spirit AI/AN	Measure name: CTQ-SF Operationalization: Individual ACE ACE prevalence: 88.1% Men and 90.6% Women reported 1+ACE	Outcome: Alcohol Misuse Measure: MINI-5, AUDIT Analysis: Logistic Regression	Experiencing childhood physical neglect and emotional abuse were associated with increased risks of past-year drinking binge or spree for two or more days. Being adopted was significantly associated with decreased risk of past-year drinking binge or spree for two or more days. For men, but not women, attending an Indian boarding school and being placed in foster care were associated with an increased risk of past-year alcohol dependence, past-year hazardous and harmful alcohol use, and past-year drinking binge or spree for two or more days. Past-year alcohol dependence was not significantly associated with total number of ACE.
*Substance Use Disorder*				
Batchelder et al. (2021)	Study design: Cross-sectional Sample Size: 263 Population: Gay and Bisexual men	Measure name: Investigator created Operationalization: CSA exposed ACE prevalence: 100%	Outcome: AUD; SUD Measure; DSM-IV AUD and SUD criteria Analysis: Logistic Regression	In unadjusted models, bisexual men with a history of CSA had an increased odds of AUD or any SUD/dependence compared to gay men with a history of CSA. In adjusted models, bisexual men with a history of CSA were only at a higher odds of stimulant use disorder.
Evans-Polce et al. (2020)	Study design: Cross-sectional Sample Size: 35,796 Population: US Heterosexual and LGB Adults	Measure name: Investigator created Operationalization: Cumulative ACE ACE prevalence: M = 4.35 (LGB)	Outcome: AUD, TUD and comorbid psychiatric disorders Measure: AUDADIS-5 Analysis: Logistic Regression	Reporting more ACEs was associated with comorbid AUD and mood disorders, AUD and PTSD, comorbid TUD and anxiety disorders, mood disorders, and PTSD but not comorbid AUD and anxiety disorders.
McCabe et al. (2020)	Study design: Cross-sectional Sample Size: 36,309 Population: US Heterosexual and LGB Adults	Measure name: NESARC-III survey battery Operationalization: Cumulative ACE ACE prevalence: SM women, M =3.0; SM men, M= 2.75	Outcome: SUD, AUD, TUD Measure: AUDADIS-5 Analysis: Linear Regression	Rates of comorbid SUD and mental health (MH) disorders were associated with reporting increased numbers of ACEs. In moderated analyses, a curvilinear relationship between the number of ACEs and comorbid SU/MH disorders was observed with bisexual and lesbian or gay subgroups being at higher risk than heterosexual subgroups.
McCabe et al. (2022)	Study design: Cross-sectional Sample Size: 36,309 Population: US Heterosexual and LGB Adults	Measure name: Wyatt Sex History Questionnaire: CSA, CTS Operationalization: Discrete ACE, Ordinal ACE ACE prevalence: SM females, M =1.5; SM males=0.8	Outcome: AUD, SUD, TUD, ODUD Measure: AUDADIS-5 Analysis: Logistic Regression	CSA was directly associated with higher risk of AUD, TUD, ODUD, and SUD, regardless of sexual orientation. However, bisexual women were at heightened risk compared to other groups.
Wolfe et al. (2021)	Study design: Cross-sectional Sample Size: 600 Population: Transgender Adults	Measure name: Investigator created Operationalization: Dichotomous ACE prevalence: 60.5% report CPA or CSA	Outcome: Substance Use, SUD diagnosis, SUD Treatment Measure: Investigator created Analysis: Linear and Logistic Regression	Experiencing CPA or CSA was associated with substance use in an unadjusted model but the relationship was not significant in the multivariate model. There were no observed relationships between CPA or CSA and SUD diagnosis or SUD treatment.

SGM sexual gender minority, MSM men who have sex with men, YMSM young men who have sex with men, MSW&TW men who have sex with men and transexual women, SMW sexual minority women, SM sexual minority, AI/AN American Indian and Alaskan native

HMA household member alcohol use, HMD household member drug use, P/CI parent/caregiver incarceration, PIPV parental interpersonal violence, CPA childhood physical abuse, CVA childhood verbal abuse, CSA childhood sexual abuse, CEA childhood emotional abuse

CTQ childhood trauma questionnaire, CTQ-SF childhood trauma questionnaire- short form, BRFSS-ACE module behavioral risk factor surveillance system, CTS conflict tactics scale, NESARC-III national epidemiologic survey on alcohol and related conditions, ETI-SR early trauma inventory- self report shortened list

ENDS electronic nicotine delivery systems

AUD alcohol use disorder, SUD substance use disorder, TUD tobacco use disorder, ODUD other drugs use disorder

AUDADIS-5 alcohol use disorder and associated disabilities interview schedule-5, MINI-5 mini-international neuropsychiatric interview, AUDIT alcohol use disorders identification test, CUDIT-R cannabis use disorders identification-revised, SIP-AD shortened inventory of problems- alcohol and drugs scale, SAMHSA substance abuse and mental health services administration

## 3 Results

### 3.1 Study Characteristics

ACEs were the primary predictor and substance use was the main outcome of all studies included in the review. Important considerations should be made when interpreting the findings of this review. First, a combination of investigator-created, investigator-modified, and validated self-report measures of ACEs and substance use were used, and the conceptualization and operationalization of ACEs varied as a result. Studies that did not utilize previously validated measures were operationalized in the current study as investigator-created. Mattera et al. (2018), asked Latino bisexual men closed-ended questions based on definitions of statutory rape to define childhood sexual assault. The specific questions did not come from a previously-validated measure: thus, studies that did not utilize previously validated measures were operationalized in the current study as investigator-created. Investigator-modified measures relied on validated measures that underwent some form of adaptation. Bertolino et al., 2020 stated, “ACE score was dependent on the 8 subsequently defined ACE categories that were adapted from previous ACE assessments.” Second, substance use outcomes were observed over different time periods (i.e., past week, past month, past year, etc.) and patterns (i.e., frequency of use, quantity of use, negative consequences), Third, while some studies were interested in the relationship of ACE to a specific substance (i.e., marijuana), others reported general drug use without sensitivity to a specific psychoactive substance (Table 1).

Across all studies, 20% assessed the impacts of ACE individually (i.e. if each ACE was examined separately), 25% used a cumulative measure of ACE (i.e. a total score without cutoffs), 15% used discrete categories of ACE (i.e. 0, 1-3, 4+), 20% used a combination of measures (i.e., cumulative ACE/ individual ACE, individual and any ACE exposure, etc.), 15% assessed CSA Exposure, and 5% looked at a dichotomous measure of ACE (any occurrence vs. no occurrence). CSA Exposure was commonly investigated; thus, it was determined to be deserving of its own category. The population sizes ranged from 131 to 36,309, and 16 of the 20 studies focused on a single SGM group (i.e., transgender women, men who have sex with men [MSM], etc.); important considerations when determining the generalizability of these studies to all subpopulations within the SGM community. Overall, the studies included in this review aligned with previous research that SGM individuals are more likely to report ACEs compared to heterosexual peers (Corliss et al., 2002; Sterzing et al., 2016; Tomeo et al., 2001).

### 3.2 ACEs and Substance Use Frequency and Quantity

Eleven manuscripts investigated the relationship between ACEs and substance use frequency and/or quantity (Table 1). While the results of identified manuscripts generally demonstrated a positive relationship between ACEs and substance use, there was a considerable range of outcomes and pathways reported. Austin et al. (2016) observed no difference in heavy alcohol use, cigarette use, or binge drinking, by sexual orientation when adjusting for cumulative ACEs exposure among 30,401 participants in 3 U.S. states. Later the researchers suggested that due to LGB (Lesbian, Gay, or Bisexual) individuals reporting a higher prevalence of ACEs, ACEs may be an important factor in determining sexual-orientation related differences in substance use outcomes. Mattera et al. (2018), however, found no significant association between CSA and polydrug use in the previous four months in a sample of Latino bisexual men.

A subset of the included studies investigated the relationship between substance use behaviors and specific ACEs. The outcomes (types of substance use), as well as the individual ACEs under investigation varied,with instances of consensus as well as opposing results. For instance, substance abuse in the home and CPA (childhood physical abuse) were associated with an increased odds of current cigarette smoking, but food insecurity and emotional abuse were not significantly associated (Lee et al., 2020). In a different study, CPA was also significantly associated with a substance use outcome: higher prescription opioid misuse in YMSM (young men who have sex with men), but CEA (childhood emotional abuse) and CSA were not associated with any prescription or illicit drug use (Kecojevic et al., 2015). Another study among MSM (men who have sex with men) (Bertolino et al., 2020) identified specific ACEs (psychological and physical abuse, exposure to mental illness or substance abuse, and parental divorce) associated with illicit substance use; however, sexual abuse, violence toward one's mother, and familial incarceration were not.

In another series of papers, specific substance use outcomes were compared (binge drinking vs. methamphetamines use). Paul and Monahan (2019) observed a difference in methamphetamine use between maltreated and non-maltreated sexual minority adults. However, this relationship was not observed for prescription drug misuse, steroid use, marijuana use, cocaine use, hallucinogen use, or days intoxicated. A study using an ordinal operationalization for ACEs observed transmasculine adult participants who reported 4+ ACEs were over 4 times more likely to report substance use compared to those reporting 0-1 ACEs. But reporting ACEs was not associated with binge drinking or cigarette use (Suarez et al. 2021). In one study investigating a highly specific SGM population (transgender women of color living with HIV), those exposed to CSA were 10 times more likely to, both, use drugs and to use a larger number of drugs compared to transgender women with no CSA history (Reback et al., 2017). A review that investigated ACE and a specific substance use outcome by geographic region (rural vs. urban Oklahoma) demonstrated that although there was no difference in ACE prevalence between the groups, ACE exposure was associated with marijuana use among urban MSM, but not rural MSM (Giano et al., 2019).

Two studies investigated the relationship between cumulative ACE exposure and substance use. Grigsby et al. (2021b) showed that for each additional ACE reported there was an increase in odds of cigarette, e-cigarette, and dual use among SGM college students. Specific to this study, ACE exposed SGM college students had a significantly higher prevalence of past 30-day cigarette use compared to non-SGM peers. Scroggs & Love (2022) observed weak pairwise correlations between ACE scores and cannabis, tobacco, alcohol, opioid, heroin, hallucinogen, and amphetamine use. The authors also created latent profiles based on levels of adversity (using an ACE score), self-esteem, hope, and community connection, showing that there was an interaction of environmental and self-concept that affected one's use of drugs. The “disconnected and high adversity” group had significantly higher rates of substance use compared to other groups.

### 3.3 ACEs and Substance Use Problems and Substance Misuse

The present review identified four publications evaluating the relationship between ACE and substance use problems or substance misuse in the SGM population (Table 1). Again, the ACEs and specific substance use outcomes investigated varied, demonstrating a complex interaction between SGM identity, life experiences, and substance use behaviors. Within a population of women, childhood victimization was found to not be associated with higher odds of hazardous drinking; however, those who were exposed to both childhood and adult victimization experienced a compounding effect and were 2.2 times more likely to experience hazardous drinking (Hughes et al., 2014). Hall et al. (2021) reported a significant interaction between teenage forced penetration and pre-teenage forced touching with higher odds of alcohol-related problems. Teenage forced penetration and teenage forced touching were associated with higher odds of stimulant use in the same study. Interestingly, participants who reported both pre-teenage penetration and teenage touching were less likely to report cannabis use problems compared to those who did not experience CSA.

In a sample of Urban Two-spirit American Indian and Alaska Native people, Yuan et al. (2014) demonstrated that different types of victims and victimization resulted in different patterns of substance use, both increasing and decreasing risks of alcohol misuse. Important to note, Yuan et al. (2014) evaluated childhood maltreatment with the short form Childhood Trauma Questionnaire and “out of home placement” with investigator-created questions. In this review the out of home placement was not evaluated due to it not being a household ACE measure laid out by Felitti et al., 1998. Overall, no strong associations were found between childhood maltreatment and alcohol misuse. There was a stronger association when evaluating out of home placement. However, women in the study who experienced childhood physical neglect or childhood emotional abuse were at increased risk of past-year drinking binge or spree. Additionally, the number of ACEs in a dose-response comparison was not significantly associated with past-year alcohol dependence. Finally, Sizemore et al. (2021) found, within a sample of transgender women, participants with high levels of attachment anxiety reported more substance use problems if they had a history of CSA. However, regardless of CSA history, participants with high attachment avoidance, or high in both attachment anxiety and avoidance, had high levels of substance use problems. The authors suggested that higher secure attachment scores can be a protective factor against substance misuse for those who have experienced CSA.

### 3.4 ACEs and Substance Use Disorder

This review identified five publications documenting the relationship between ACEs and substance use disorder in SGM populations (Table 1). Three studies used large samples of 35,000+ adults across the United States. The first two of these used cumulative ACE scores to investigate comorbidity of substance use outcomes and mental health disorders, importantly both studies demonstrated positive associations. Evans-Polce et al. (2020) investigated the cumulative effect of multiple ACEs and comorbidity of alcohol (AUD) or tobacco use disorder (TUD) and a psychiatric disorder. The authors found a greater number of ACEs were associated with comorbid AUD and mood disorders, AUD and PTSD, TUD and anxiety disorders, TUD and mood disorders, and TUD and PTSD, but not comorbid AUD and anxiety disorders within SGM individuals. McCabe et al. (2020) observed that reporting more ACEs was associated with comorbid SUD and mental health disorders in LGB identifying individuals. Later, McCabe et al. (2022) investigated specific substance use outcomes and their association with CSA. A heightened risk for SGM for AUD, TUD, ODUD (other drug use disorder), and SUD among those who were exposed to CSA, after controlling for other ACEs, was observed. The relationship was strongest for bisexual individuals, bisexual women in particular.

The last two studies investigated a specific SGM population and specific ACEs with more general substance use disorder outcomes. In an unadjusted regression model of 600 transgender adults, Wolfe et al. (2021) reported that experiencing CPA or CSA was associated with substance use, though the effect dissipated in the adjusted model (including age, gender identity, race, survey modality, discrimination attributable to other reasons than being transgender, CPSA, and IPV). Additionally, there was no association between CPA or CSA and a SUD diagnosis or undergoing SUD treatment. Batchelder et al. (2021) found more bisexual men with a history of CSA to meet criteria for AUD and other substance use disorders than gay men without a history of CSA; however, these differences disappeared when race, education, and income were included as covariates. Adjusting for covariates, CSA-exposed bisexual men were the only subgroup at a higher odds of meeting criteria for the specific diagnosis of stimulant use disorder.

## 4 Discussion

This review identified a growing and diverse number of studies examining the relationship between ACE and substance use among SGM populations in the years following the Schneeberger et al. (2014) review. In line with the results of Schneeberger et al. (2014), this review identified relationships between exposure to childhood sexual abuse and multiple substance use outcomes. However, this review builds on previous findings by identifying relationships between ACEs and SUD across different SGM populations (Two-Spirit American Indian and Alaskan people, Transgender women of color living with AIDS, etc.) as well as differences that exist between rural and urban contexts, for new substance use outcomes (i.e., ENDS), and by other demographic characteristics (e.g., sex assigned at birth, race/ethnicity, and between sexual and gender minority subpopulations). This review also highlights the wide range of investigation of substance use behaviors, including frequency & quantity, substance use problems and substance misuse, to clinically relevant substance use disorders.

Overall, exposure to ACEs was positively associated with substance use, across sexual- and gender- identities, aligning with previous research (Douglas et al., 2010; Irwin, 2017; Scheeberger et al., 2014). However, it is notable that studies investigating relationships between individual ACE experiences and substance use produced more significant relationships than those investigating the impact of cumulative ACEs (Bertolino et al., 2020). This suggests that beyond total trauma exposure, experiencing specific traumatic events in childhood may uniquely explain substance use behavior in adulthood for SGM populations. Of course, many subgroups of the SGM community were sampled across studies making comparisons difficult. Further work with large SGM samples is needed to uncover patterns of ACE exposure and substance use behavior between sexual and gender identities. Nevertheless, the research to date suggests that SGM adults with a history of ACEs should be considered a priority population for substance use prevention and intervention programs.

Few studies have investigated relationships between multiple ACEs and substance use problems in SGM samples, and most focused on the relationship with childhood sexual abuse experiences. Taken together, CSA is positively associated with experiences of substance use problems which is consistent with work among the general population (Nelson et al., 2006; Wilsnack et al., 1997). As suggested by Wilson (2010), the health consequences of CSA are broad and varied. It must be emphasized that the trauma continues after the event as individuals deal with shame, regret, anger, and other issues. Interdisciplinary and concerted efforts are needed to develop preventive interventions that address the CSA event and sequelae of events that can have a detrimental impact on adult health status and functioning. One study (Hughes et al., 2014) observed combined effects of experiencing trauma from childhood into adulthood on hazardous drinking behavior. This finding suggests developmental timing and chronicity of ACE experiences may be an important predictor of adult health outcomes, including substance use and misuse.

SGM populations were also at an increased risk of multiple substance use disorders following exposure to ACEs. Results of nationally representative analyses suggest bisexual individuals, in particular, are especially vulnerable to the effects of ACEs–a finding that aligns with previous work regarding notable health disparities for bisexual adults (Friedman et al., 2014). It remains unclear why this subpopulation is at heightened risk for SUD following ACE exposure. While the impact of other forms of trauma (i.e., discrimination, generally, and internalized homophobia, specifically) have been attributed to sexual orientation (Kim & Fredriksen-Goldsen, 2012), it remains unknown whether bisexual orientation is involved in the etiology of ACE exposure or whether other traumatic events, not captured in the included studies, produced an additive or multiplier effect on ACE and SUD. Future research is needed to understand why ACE exposed individuals who identify as bisexual are at heightened risk for SUD compared to other SGM populations.

### 4.1 Limitations

This review is not without limitations, and the results should be interpreted with the following in mind. First, we limited the timeframe of the search from November 2021 and February 2022 using five databases, and there is a possibility that relevant literature was not captured. Second, our search terms were designed to measure the impact of childhood adversity in the home and cannot be generalized to community or school experiences such as bullying, structural discrimination, or witnessing violence toward SGM groups, for example. This is an important consideration given that SGM youth are likely to experience adversity in multiple contexts due to heterosexism, and points to the need for an intersectional SGM-ACEs framework (Schnarrs et al., 2022). Third, the present review focused on substance use and related outcomes, misuse and substance use disorder, and cannot be extrapolated to explain other negative health behaviors. Lastly, we limited our search to studies published in the United States thus limiting the generalizability of these findings to global sexual and gender minority individuals.

The findings of this review also revealed several existing limitations in the literature that should be addressed in future research. First, all but one study (Paul and Monahan, 2019) used a cross-sectional design which limits our ability to draw cause-and-effect conclusions regarding observed relationships. Second, several studies investigated a single type of ACE, and among those, the majority focused on childhood sexual abuse. A vast literature has documented the correlated nature of ACEs and that individual ACEs have a cumulative impact on health when modeled simultaneously (Rogers et al., 2022). Moreover, researchers should be mindful of selecting validated instruments with more exhaustive lists of ACE events such as the Childhood Trauma Questionnaire (Bernstein et al., 1997) or the ACE-International Questionnaire (Ho et al., 2019), and measures that assess unique ACEs experienced by SGM individuals should be considered as well, such as the SGM-ACE (Schnarrs et al., 2022). Third, substance use frequency was investigated far more often than substance use quantity; however, frequency was assessed with myriad questions covering a variety of timeframes (past week, month, year, etc.). Similar to measuring ACEs, substance use behavior should be assessed using a standardized approach such as the Timeline Followback Survey (TFBS; Sobell et al.,1996) in order to capture both frequency and quantity of use simultaneously. Finally, the majority of existing research has been carried out with sexual minority populations with much less attention given to gender minority groups. More work is needed to understand the relationship between ACEs and substance use behavior in this population.

## 5 Conclusion

Experiencing ACEs has a detrimental impact on the substance use behavior of SGM populations and trauma-informed interventions are needed to offset the potential life-course harms of childhood maltreatment.

## Contributors

BD, GZ, and TG reviewed articles and abstracts and BD coded results. BD and TG drafted the initial manuscript. BD, TG, GZ, and PS edited and contributed to the writing in subsequent drafts. All authors have substantially contributed to the data collection or manuscript development. All authors have approved the final article should be true and included in the disclosure.

## Declaration of Competing Interest

No conflict declared

## Appendix Supplementary materials

Image, application 1
