# Supplementary material for: Substance Use Outcomes For Sexual and Gender Minority Adults With a History of Adverse Childhood Experiences: A Scoping Review
Source: Drug Alcohol Depend Rep. 2022 Dec 9;6:100129. doi: 10.1016/j.dadr.2022.100129 (PMC10040327; doi:10.1016/j.dadr.2022.100129)
Supplement: Supplementary file 1 [file mmc1.docx]

Results for TS=(alcohol OR tobacco OR cannabis OR club drug OR hard drug OR illicit drug OR prescription drug misuse OR problem substance use OR substance related negative consequences OR drug abuse OR drug dependence OR substance-related disorders OR substance use disorders) AND TS=(domestic abuse OR physical abuse OR sexual abuse OR neglect OR adverse childhood experiences OR childhood maltreatment) AND TS=(LGBT OR gay OR lesbian OR transgender OR bisexual OR sexual diverse OR sexual minority OR gender minority) and Review Article (Document Types) and USA (Countries/Regions) and Humans (MeSH Headings)

Date Range: 2014-01-01 to 2022-01-01
